# Can Potted Plants Catch Mosquitoes? Applying Rare-Earth Luminescent Materials and Plant Energy to the Development of Innovative Mosquito-Trapping Potted Plants

**DOI:** 10.3390/ijerph20043368

**Published:** 2023-02-14

**Authors:** Lung-Yin Lin, Yu-Shing Chang

**Affiliations:** 1 Department of Industrial Design, National Kaohsiung University of Science and Technology, No.1, University Rd., Yanchao Dist., Kaohsiung City 82445, Taiwan; 2 Department of Business Administration, National Taichung University of Science and Technology, No. 129, Sec. 3, Sanmin Rd., North Dist., Taichung City 404, Taiwan

**Keywords:** energy-saving design, patent, rare-earth luminescence, plant energy, epidemic prevention

## Abstract

Due to the global warming crisis, the spread of various infectious diseases is worsening, with mosquito-borne contagious diseases posing a significant threat. While many residential and public spaces contain plants, often for greening the environment and improving mental and physical well-being, the carbon dioxide released by these plants produces ideal habitats for mosquitoes. Considering the quality of life of urban residents and the development of health-related products simultaneously is an important topic. This study used diverse complementary techniques, such as energy-storing rare-earth luminescent materials, sustainable power generation using plant energy, blue light–emitting diodes, and environmentally friendly fermentation formula, to develop planting products with potential mosquito control functionality. The prototype design for this mosquito-trapping potted plant has been patented. The aim of this paper is to discuss the design principles adopted to improve the defects of existing mosquito-trapping designs, the green energy materials and techniques employed, the architecture configuration of the product prototype, and the test results. By integrating green materials and technology, the prototype can be self-powered without being plugged in to yield conspicuous energy savings. The results showed that the developed multi-function products, combined with the concept of energy sustainability, can improve global public health as well as individuals’ physical and mental health.

## 1. Introduction

Owing to global warming, many infectious diseases are spreading more quickly, resulting in increasing rates of mosquito-borne contagious diseases such as dengue fever, malaria, Japanese encephalitis, and Zika virus. Among these, dengue fever poses a severe health risk [1]. As estimated by the World Health Organization (WHO), there are approximately 390 million cases of dengue fever every year. Its prevalence increased by 15 times over the past 20 years, while the death toll has increased by four times. Climate change-related variations in mosquito population dynamics have allowed dengue fever to spread from low latitudes to middle and high latitudes. It is now prevalent in 129 countries in Africa, America, Eastern Mediterranean, Southeast Asia, and the Western Pacific, with 70% of cases being reported in Asia [2]. According to a study by Shepard et al. (2016) evaluating 141 countries with active dengue transmission, 18% of all cases were hospitalized and 48% received outpatient treatment, leading to an annual financial burden of USD 8.9 billion [3]. In India, the annual economic and social cost of dengue fever was determined to be USD 1.13 billion, which is comparable to the nation’s annual expenditure on space research and development. In Kerala, for example, the costs of disease treatment, prevention, and death associated with dengue fever are conservatively estimated at USD 38 million, with death-related costs being the highest at USD 27.7 million. Brazil’s annual expenditure on epidemic prevention is approximately USD 1.23 billion. Thailand’s annual tourism revenue decreased by USD 370 million during the dengue pandemic. In Malaysia, parents who had one child hospitalized with dengue fever lost at least 16–23 working days [4,5]. In recent years, the COVID-19 pandemic has placed immense pressure on healthcare and management systems worldwide. The WHO has emphasized that the combined impact of the COVID-19 and dengue epidemics may have devastating consequences on at-risk populations [2]. These statistics show that mosquito-transmitted disease not only has a great impact on public and individual health globally but also leads to significant economic and social burdens worldwide.

In cities with large populations, residents attach great importance to the green environment [6]. Green environments have led to improved air quality [7], reduced carbon emissions [8], regulated temperature and humidity [9], provided animal habitats to promote ecological diversity [10], beautified the city, promoted balance in residents’ physical and mental states [10,11], and improved happiness and life satisfaction [12]. However, the carbon dioxide released by photosynthesis within green environments can lure mosquitoes. Indeed, there is a positive correlation between the area of green space and the number of mosquitoes in cities [13]. Most relevant studies investigating the impacts of climate change on health risks for urban residents have considered intervention strategies that may improve residents’ resilience to climate change and evaluated the effectiveness of those strategies [14,15,16,17,18]. Many of these studies have employed macroscopic (i.e., national or city level) assessment adaptation strategy tools, while few studies have proposed designs for epidemic prevention interventions on a microscopic (i.e., household) level. Sahay described the national-level disease control plans that India has adopted against the mosquito vector [18]. These plans involved establishing designated monitoring hospitals and referral centers for early case reporting and management, vector management including anti-larval and anti-adult measures, and supporting interventions such as capacity building. The household-level coping strategies focused on preventing mosquito biting and breeding by using insect repellents and mosquito nets and cleaning flowerpots and areas prone to water accumulation. It has been shown that anti-mosquito measures taken by residents are significantly more effective than government interventions, highlighting the importance of home mosquito control products. Nevertheless, most studies have focused on the performance of mosquito products designed for use on farms or in fields [19,20,21,22,23,24]. Despite the known importance of micro-level interventions, there has been limited research on the functions of mosquito products designed for household use. Hence, this is an issue worthy of further investigation.

Experts have proposed various methods to combat mosquito infestation, such as long-lasting insecticidal nets and indoor residual spraying. However, these are not suitable measures as mosquito nets do not affect outdoor spaces, while mosquito repellents (such as coils, mats, and liquids) are typically harmful to the human body. Moreover, mosquito populations are prone to developing drug resistance [25,26,27]. Recently, with the rise in public health awareness, natural anti-mosquito products have gradually replaced mosquito coils and insecticides that use chemical mosquito repellent components [28]. Physical methods such as light, color, and heat have also been used to trap mosquitoes [22]. For example, light traps are commonly used, and effective mosquito traps are available on the market [29]. Industrial research determined that the market opportunities for light traps will continue to increase until 2026 [30]. However, light traps have several disadvantages, including power consumption, visual and auditory disturbance, location limitations due to power socket requirements, and having a single function rather than multiple ones [31]. Global low-carbon energy transformation is driving the green economy of urban spaces and adapting to the sustainable trend of green materials and energy while considering the issues of environmental beautification and mosquito epidemic prevention is vital in innovative product design [32].

Through a literature review, we identified that the rampant dengue fever epidemic due to global warming has posed a significant global social and economic burden and that the COVID-19 pandemic has highlighted the importance of comfortable, green (household) environments for improving individuals’ physical and mental well-being. However, the green environment is prone to facilitating mosquito habitat formation. Most previous research discussed the prevention of mosquito transmission from the general policy aspect, and few studies have addressed the perspective of strengthening prevention efforts through the design of individual prevention products. Moreover, the research on anti-mosquito lamp design mostly addresses farm or field scenarios but not the household environment. Therefore, this study aims to address the shortcomings of previous research and mosquito control products and design an innovative green energy mosquito-trapping potted plant for home use that can help cope with global warming and the dengue fever pandemic, reduce economic and social costs, green the environment, and improve the physical, mental, and spiritual wellbeing of urban residents through expert interviews, technical knowledge, and integrated applications of green energy materials.

## 2. Material and Methods

In the process of designing this mosquito trap, a literature review was conducted, and four Taiwanese scholars and experts were interviewed to discuss the challenges pertaining to mosquito habits and trapping techniques. These discussions were very informative and enlightening.

We thoroughly investigated different rare-earth luminescent materials mixed with different ratios of composite materials to determine their suitability for our mosquito trap. Mosquito lure formulations were tested with varying contents at different proportions. The operating model and prototype performance are presented.

### 2.1. Product Design Concept

Mosquito-trapping products for human comfort and health and planting products for environmental beautification appear to serve independent and specific functions. However, these products can create usage conflict because plants for beautification can create damp, dark areas and release carbon dioxide, which can promote inhabitation by mosquitoes. Therefore, product designs that simultaneously consider environmental greening and mosquito epidemic prevention, while conforming to global energy-saving and carbon reduction trends, should be considered.

Mosquitoes are known to be attracted to light, carbon dioxide, humidity, and lactic acid smells [21,33]. Thus, most commercial mosquito-trapping products make use of light and carbon dioxide production to trap mosquitoes [22,33]; however, these methods can give rise to glare and noise, which can cause discomfort in humans. Furthermore, mosquito traps are mainly powered by electricity and are limited to locations with suitable power supply, thus coming into conflict with the goals of environmental sustainability and greening. These products also require consumers to manually switch on the power supply and only provide a single function, thus reflecting a lack of functional diversification and human factor design principles. Therefore, in this study, the trapping methods, energy modes, switching modes, usage modes, and multi-purpose effects of commonly used mosquito-trapping products on the market were analyzed to provide a direction for innovative product design.

### 2.2. Materials and Technologies Used

The characteristics and objectives of the materials and technologies used in the product design process are described below and summarized in Table 1.

#### 2.2.1. Rare-Earth Self-Luminous Mosquito Luring

Rare-earth luminescent materials provide green lighting with less pollution and are widely used in warning sign systems at construction sites, expressways, urban streets, airports, ships, and hospitals [34,35,36,37]. The advantages of rare-earth luminescent materials include stability, high-temperature resistance, highly pure and bright colors, high light conversion efficiency, and strong absorptive capacity [36,38,39]. Rare-earth luminescent materials can store energy during the day and emit light at night or under low-light conditions. Research has shown that light has a good mosquito-luring effect. Among the light sources used in such applications, UV fluorescent, blue and green LEDs have the best effect in luring mosquitoes [21,22,40,41]. However, the difference in effectiveness between blue and green LEDs in luring mosquitoes is controversial. Some studies have concluded that blue LEDs have better mosquito trapping performance on average [33], while other studies have concluded that green LEDs yield better performance [21]. Costa-Neta et al. contended that green LEDs used in previous experiments were 9000 mcd stronger than blue LEDs (green = 15,000 mcd; blue = 6000 mcd). 6000 mcd, which may be biased because the potential effect of luminous intensity on mosquito trapping performance was not considered [22]. Light-storing rare-earth materials conventionally put off green and blue light, and green- and blue-luminous materials are cheaper and easier to obtain than purple-luminous materials. Moreover, consumer perception is not good toward green light in the household environment, so there are few commercially available green light anti-mosquito lamps. After comprehensive consideration, this study employed a blue-light rare-earth material with blue LED to achieve both mosquito-trapping effects and cost-effectiveness. In addition, due to the high cost of rare-earth luminescent materials, ensuring an effective proportion of energy storage luminescent materials within composite materials is crucial to their luminosity and cost. Rare-earth luminescent materials have never been used in mosquito luring design, and this study is the first to employ blue-light rare-earth materials to improve mosquito luring in a trap design. The constituent material ratios and casting shape were optimized to reduce costs while ensuring effective luminosity.

#### 2.2.2. Photoresistor and LED Light to Lure Mosquitoes

Previous research has shown that LEDs have a good mosquito-luring effect [21,40,41]. In addition, the photoelectric control circuit can be switched on and off based on the presence of light through the photoresistor to reduce power consumption and limit user effort. The photoresistor also has the characteristics of high sensitivity, response speed, stability, and reliability [42]. Therefore, combining the photoresistor and LED to automatically switch the LED light on and off between day and night to lure mosquitoes is a design that considers the mosquito-killing effect and user friendliness. LED lighting can also assist in prolonging the luminous effect of rare-earth materials. In addition, the photoresistor can be programmed to set the light-emitting time of the LED. For example, if the electricity storage LED modules are set to rest for two hours every three hours, the rest of the time can be supplemented with rare-earth materials, which can further save energy.

#### 2.2.3. Plant Energy for Sustainable Power Generation

In general, light traps need power connection continuously to ensure that people are not troubled by mosquitoes when doing activities or sleeping, making them inconvenient to place outdoors due to the lack of electricity. Moreover, the power consumption requirements of these products make them not environmentally friendly. During routine watering and photosynthesis in plants, a potential difference is formed through the electrolytic reaction of copper and zinc metal sheets upon contact with water, which generates electricity. The organic matter formed by plant photosynthesis and the effects of fertilizer and soil pH leads to the release of positive and negative ions, enhancing power generation. In recent years, this mode of power generation has gained attention in European countries. For example, the Netherlands has been committed to integrating natural energy and design and developing lighting applications related to plant power generation [43,44]. Therefore, this research adopts plant energy for sustainable power generation in the product design.

#### 2.2.4. Principles of Environmental Protection and Mosquito Killing

With increasing health awareness, natural anti-mosquito formulas have gradually replaced chemical components. Additionally, research has confirmed that mosquitoes are drawn to carbon dioxide and acid odors [33]. Therefore, a nontoxic and harmless fermentation formula was designed to release carbon dioxide and a slightly acid fermentation odor to lure and kill mosquitoes.

#### 2.2.5. Innovative Integrated Design

To allow the potted plants used in the design to be changed according to the user’s wishes or seasons while considering beautification, variability, and decoration, the plants were independently potted within an organizer box that can be removed and replaced at any time and can be arranged and combined in different ways. The greening effects of potted plants and mosquito-trapping functions were integrated for the innovative design of a value-added product. This design allows creativity and diversity while supporting epidemic prevention, beautification, and environmental protection.

## 3. Product Design

### 3.1. Product Technology Principle, Operation Process, and Function Configuration

**Figure 1:**
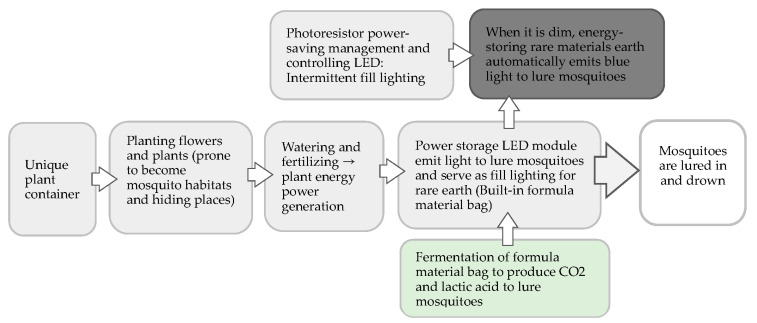
Technological principles and operation process of innovative green energy mosquito-trapping plant potting technology.

**Figure 2:**
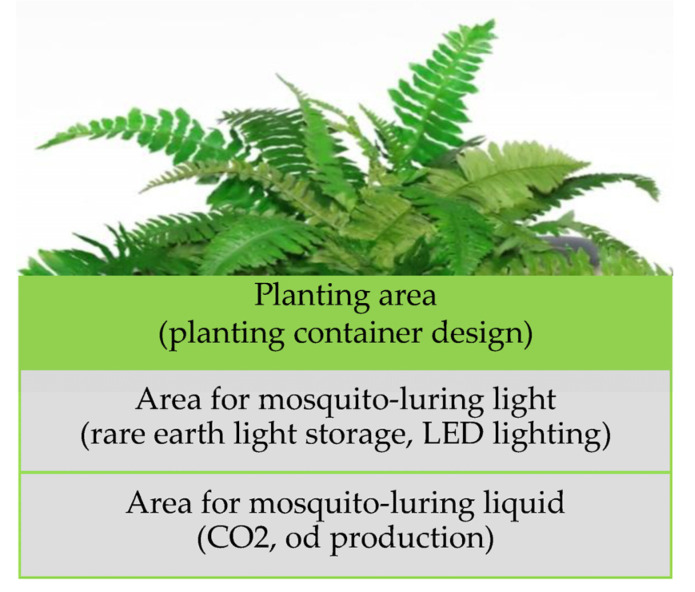
Schematic of innovative green energy mosquito-trapping potted plant products.

The above design concepts were used as the design inputs. From these, the initial concept of the product design was designed to serve as a reference for refined prototype function and production of subsequent products through a literature review, expert interview, and available materials and technology exploration. Its technology principle, operation process, and function configuration concept are presented in Figure 1 and Figure 2.

### 3.2. Product Design Principles and Prototype

The product design principles to achieve green electricity usage and mosquito luring were mainly divided into four parts: (1) The mosquito trapping area is equipped with energy-storing rare-earth and electricity storage LED modules (storage battery, printed circuit board, LED). Two UV LED bulbs are combined with energy-storing rare earth materials and placed in a protective mask to lure mosquitoes; the latter can absorb daylight and emit blue light. In the evening, when the LED is on, the system can lure mosquitoes, and the energy supply is supplemented with the energy stored by rare-earth materials. The electricity storage LED modules automatically switch the blue LED using the photoresistor to lure mosquitoes at night. In addition, the photoresistor can be programmed to set the light-emitting time of the LED to further save energy. The Y-shaped seam above the removable drawer is similar to the funnel, with a large opening and a small exit that was easy for mosquitos to enter but difficult for them to escape; (2) opper and zinc sheets are arranged in this section. This design releases positive and negative ions from the copper and zinc sheets in the soil to generate electricity during routine watering and fertilizing actions from the user; (3) The bottoms of the potted plants have a removable drawer, convenient for replacing the new natural fermentation formula bag, which is responsible for releasing carbon dioxide and luring mosquitoes to the acidic odor; (4) the unique plant containers can be replaced with other plants at any time according to the season and user preferences.

The prototype was designed by three Dimensions (3D) computer drawing and configuration, and the prototype materials were selected based on established commercial products. The plastic components such as the exterior, extractable potted plant box, mosquito trapping drawer, and power storage module area are mainly made of Acrylonitrile Butadiene Styrene (ABS) through Computerized Numerical Control (CNC) milling. The electronic parts such as copper and zinc plates, storage batteries, LED and photoresistors, and PCB modules (including CPU and resistor) were purchased as they are all commercially available parts.

The control parameters of the power storage time of the power storage LED module and the discharge time of the photoresistor were programmed into the CPU through power management and sensing parameters. Through consumers’ daily planting actions such as watering and fertilizing, the positive and negative ions from the decomposition of copper and zinc plates in the soil will be received and stored by the storage battery. The storage effect varies depending on the type of fertilizers applied and the pH value of the soil. The photoresistor detects the brightness of the environment to either illuminate or turn off the LED like how intelligent night lights sense light/dark conditions to actively switch on and off. This saves power and is user-convenient, as the photoresistor only turns on the LED power when the environment is sufficiently dark. Furthermore, for a greater power-saving effect, the parameters of the time at which the photoresistor turns on/off power can be included in control programs to meet specific user needs.

**Figure 3:**
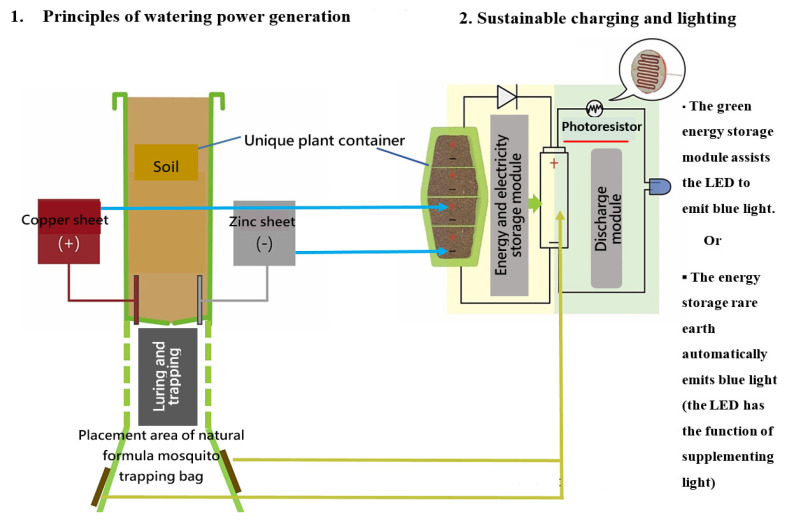
Design principles of innovative green energy mosquito-trapping potted plants.

**Figure 4:**
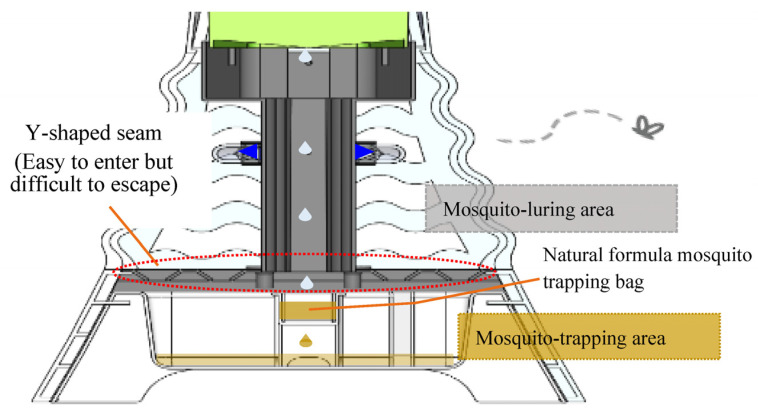
Mosquito luring/trapping area cross-section.

**Figure 5:**
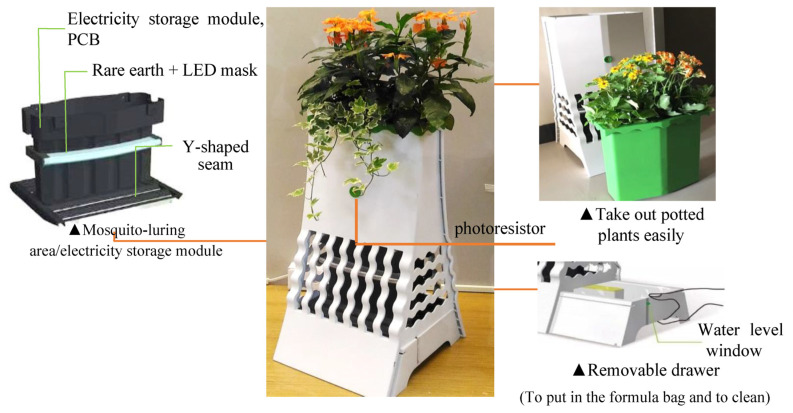
Innovative green energy mosquito-trapping potted plant prototype.

By integrating these innovative and complementary design principles, this design achieved the three functions of sustainable charging and lighting, health protection and epidemic prevention, and space greening. The detailed design principles and prototype are shown in Figure 3, Figure 4 and Figure 5. The prototype has been granted a utility model patent by the Intellectual Property Office of the Republic of China (No. M565961).

## 4. Experimental Results and Discussion

### 4.1. Rare-Earth Composite Material Formula and Plant Energy Power Generation Experiment

The blue light-emitting rare-earth material must be castable after mixing without exhibiting color distortion. Moreover, the materials must be easy to obtain. Epoxy Resin AB Glue (Epoxy AB) and poly are the only two suitable materials that currently can be mass-produced. According to the experimental results of previous studies, the rare-earth material with composite material is epoxy AB. According to the results of previous experiments, a good luminescence effect and cost-effectiveness can both be achieved when the rare-earth material with composite material epoxy AB is used at a ratio of 35:65% [39]. Moreover, the light storage powder tends to remain in a particulate state and cannot be completely dissolved at a ratio of over 40%.

Therefore, we mixed blue-light-emitting rare-earth materials with composite material epoxy AB in 3:7 and 4:6 ratios and tested the performance of the mixtures, which were shaped into upside-down cones with base areas of 50 mm^2^ and heights of 5, 10, or 15 mm. Their luminescence intensities under indirect daylight and indoor lighting were measured.

**Figure 6:**
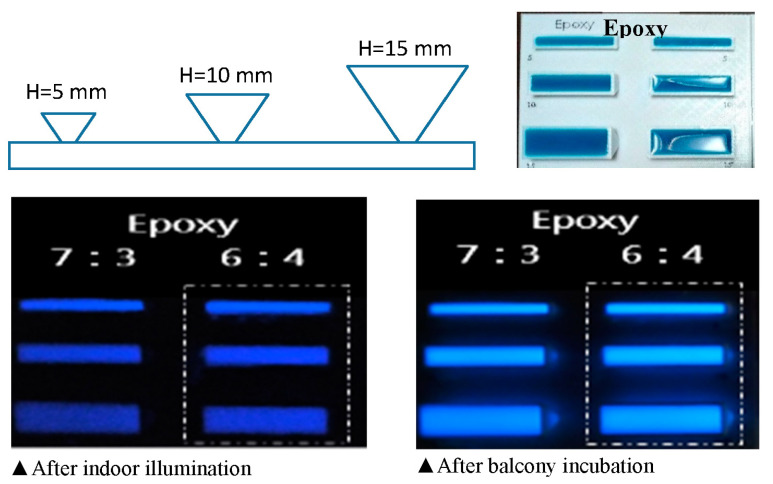
Comparison of light intensities for different ratios of solvent to long-acting rare-earth luminescent material applied indoors and on a balcony.

Previous studies have reported better mosquito trapping performance for mixtures with stronger luminescence intensity [22,23]. The mixture produced a cone with a height of 15 mm. A mixture of 40% rare-earth material and 60% Poly generated the strongest luminescence intensity, as shown in Figure 6. The longest self-luminous time of third-generation light-storing rare-earth materials is 12 h (high-efficiency type). General light-storing rare-earth materials have achieved self-luminous times of 4–4.5 h. The rare-earth material in the 4:6 mixture weighs less than 10 g and costs approximately USD 1.60, making it quite cost-effective. Therefore, the rare-earth material in the 4:6 mixture with a height of 15 mm was used as the reference for the mosquito-trapping design.

**Figure 7:**
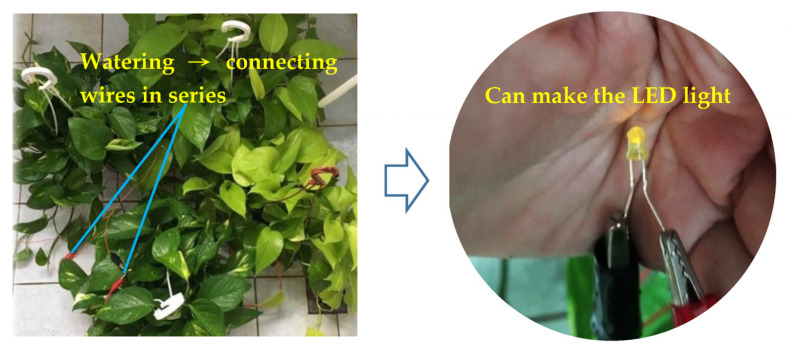
Effect of plant energy power generation.

Regarding the effect of plant energy power generation and energy storage, copper and zinc sheets are wired into the soil, and the added water decomposes positive and negative ions under the fertilizer pH. Then, they are connected in series to the positive and negative LED poles to illuminate the LED, as shown in Figure 7. Power generated by the plant was connected to the power storage module, which contains two AA rechargeable lithium batteries connected in parallel with the PCB to receive photoresistive signals to control whether to turn on/off the LED lights and to control the ions driven by water poured to store power in the batteries.

### 4.2. Proportions of Ingredients in Mosquito-Trapping Formula

Data collection was conducted to obtain effective and low-cost natural mosquito-trapping formulas following the suggestions of experts. The formulas can be roughly divided into two major types: fermented sugar water and sweet and sour solution [45,46,47].

**Figure 8:**
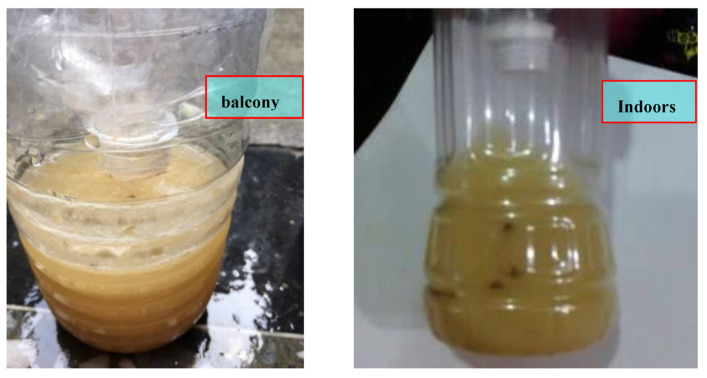
Mosquito catching result using formula C.

As shown in Table 2, experiments were carried out using six formulas (A–F) with different ingredients and proportions, and the anti-escape mechanism was established by placing a polyethylene terephthalate (PET) bottle upside down to form a large opening and small exit, thus making it easy for mosquitos to enter the bottle but difficult to exit (Figure 4 and Figure 5). Experiments were carried out indoors and on a balcony within a 24 h period on 30 November 2021. Formula C exhibited the best mosquito-trapping effect, trapping five and seven mosquitoes indoors and on the balcony, respectively (Figure 8), while the other formulas trapped 2–4 mosquitoes indoors and on the balcony. Therefore, formula C was used as a reference for subsequent formulations. In addition, formula C was considered to be effective for at least ten days according to the experts’ experience, meaning that the mosquito-trapping bag only needs to be replaced every ten days.

### 4.3. Operating Model and Prototype Effects

**Figure 9:**
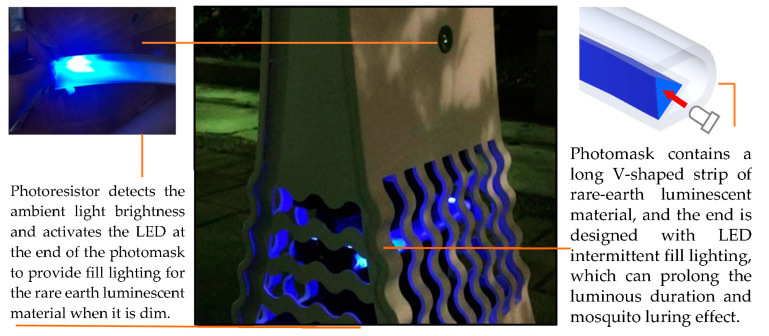
Innovative green energy mosquito-trapping potted plant prototype at night.

The product prototype (Figure 5) was developed based on the operating principles and configuration framework presented in Figure 3 and Figure 4. The electricity storage module, which combines rare-earth materials and plant energy, emits light at night through the photoresistor LED. The operation details are as follows: a V-shaped long strip of blue-light rare-earth luminescent material was poured into the photomask. The LED was set up at the end and used in combination with photosensitive resistors to detect ambient light conditions. The LED can be activated as an intermittent fill lighting for the rare-earth material when it is dim and can thus extend the luminous duration and mosquito-luring effect (Figure 9).

**Figure 10:**
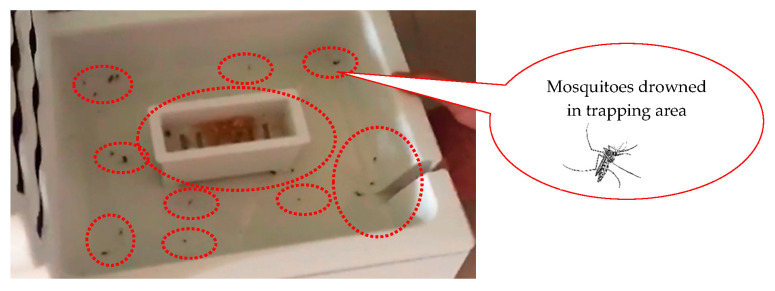
Prototype mosquito-trapping effect.

The mosquito-luring fluid generates CO_2_, which lures mosquitoes during the day for 12 h without power. At midnight (12 a.m.), the photoresistor turns on the LED for three hours and switches it off for the following two hours, allowing the LED and rare-earth luminescent material to emit blue light in intermittent cycles supplemented by the mosquito-luring fluid. The prototype designed in this study was operated for a 24 h period on 15 May 2022, on a balcony. The temperature was 24–28 °C, and the mosquito-trapping effect after 24 h is shown in Figure 10. The area circled by the red dashed line presents the mosquitoes drowned in the natural mosquito-trapping formula area.

## 5. Conclusions

This study presented and evaluated an innovative mosquito trapping product design with multiple benefits. These benefits are discussed in this section.

### 5.1. Environmental Protection

The light-storing rare-earth materials used in this design are environmentally friendly and the green materials are non-toxic, sustainable, and non-radiative. The mosquito-trapping formula bag is non-toxic, harmless, natural, environmentally friendly, and free of artificial chemical components.

### 5.2. Energy Saving

Due to the self-generating design based on rare-earth materials and plant energy, the energy-saving effect of this design was conspicuous compared to that of conventional plug-in photocatalytic mosquito traps.

### 5.3. User Friendliness

The product design is considerate of human safety and comfort as it generates no noise (electric shock sound or fan sound), glare, or harmful smells and uses no artificial chemicals.

### 5.4. Convenience

The potted plant containers are movable and replaceable. The plant arrangement can be changed by the user to improve aesthetic diversity according to the season. The placement area for the mosquito-trapping formula bag was designed as a removable drawer. The mosquito luring lights are controlled by the photoresistor to automatically switch on and off between day and night for improved user convenience.

### 5.5. Health Promotion

The rational aspect of this product design has the function of killing mosquitoes for epidemic prevention while the perceptual element can green the environment, satisfy user interest in planting, enhance the aesthetic of their homes, and support users physical and mental health.

The overall design was based on the known behavior of mosquitoes, including being attracted to blue light and acid odor, in combination with cross-domain technology, human factor design, complex functions, site applicability, and convenience to simultaneously achieve environmental greening, mosquito control, and energy conservation and sustainability. The popularization and application of this design can link the environmental awareness and lifestyle of urban residents to improve daily life and well-being while supporting sustainable product development. Moreover, it serves to reduce the significant economic and social burden posed by mosquito-borne diseases worldwide.

## 6. Research Recommendations

**Figure 11:**
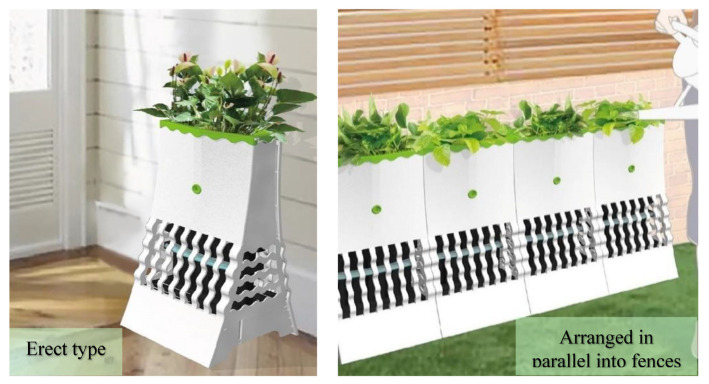
Erect-type innovative green energy technology mosquito-trapping potted plants.

**Figure 12:**
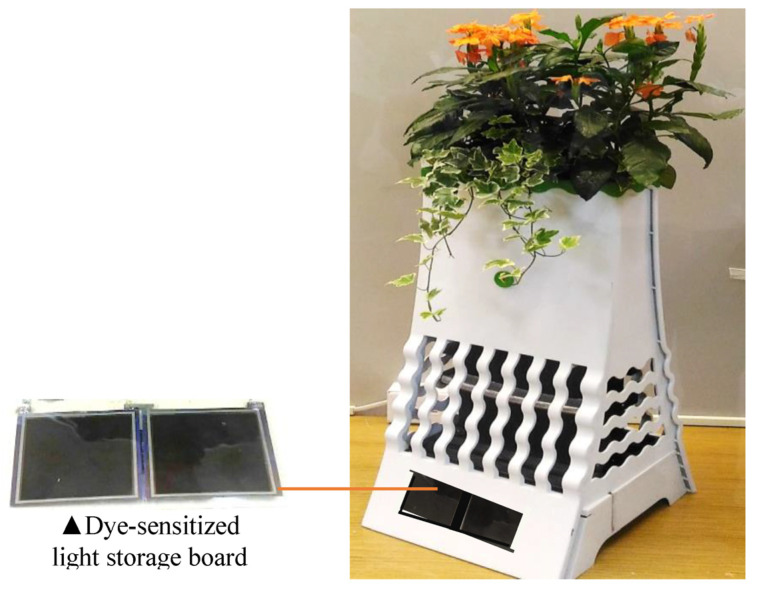
Advanced fusion innovative green energy mosquito-trap potted plants prototype with dye-sensitized solar technology.

The developed product can be placed on a balcony or by the window conveniently without being plugged in. In addition, the design can be extended to an erect type and combined into long fences to yield more diverse placement possibilities, as shown in Figure 11. This design has diverse usage opportunities and can be employed expansively with different sizes in the following places: (1) outdoor spaces: parks, community courtyards, road separation islands, courtyards, and lakesides; (2) commercial spaces: art museums, science museums, and shops; (3) and exhibition sites: exhibition venues and trade exhibitions. Sustainable energy such as solar panels for outdoor spaces or dye-sensitized solar cells for indoor spaces (as shown in Figure 12) could also be used to complement the power of the plant energy. The next step for developing this prototype is to collaborate with a product or industrial designer to design a range of appearances for the product suitable for different purposes and locations for environmental greening and supporting residents’ wellbeing. As the effect of plant energy power generation and storage may vary depending on the type of fertilizers applied and soil pH, further tests can be conducted at the commercialization stage for the sake of consumers’ reference when planting.

In addition, Sahay demonstrated that measures taken by residents against dengue fever are more effective than government interventions and that income level is the main factor affecting the tendency to adopt such measures [18]. Considering that many residents in developing countries have economic constraints, a low-cost version of the product that employs light-storing rare-earth materials and an environmentally friendly mosquito-trapping formula is expected to be useful.

## Figures and Tables

**Table 1:** Product design features and objectives.

Materials/Technology	Characteristic	Purpose
Energy storage rare earth	Energy storage during the day and self-illumination at night.	User-friendly, environmental protection, and epidemic prevention: during the day, it stores light energy and does not consume electricity and in periods with a higher number of mosquitoes at night, it spontaneously lures mosquitoes using blue light.
Photoresistor and LED light	Convertible photoelectric control LED switch.	User-friendly and energy-saving: the photoresistor automatically switches the LED on and off between night and day. In addition, it can be programmed to set the light-emitting time of the LED. Environmental protection and epidemic prevention: The energy-saving blue LED lures mosquitoes and supplements light for rare earth.
Plant energy power generation	Planting, watering, and fertilizing can generate positive and negative ions for the power supply.	User-friendliness: watering and fertilizing are routine. Energy-saving, beauty, and convenience: green energy devices can be placed indoors and outdoors without the need for power connection.
Mosquito-trapping formula	Natural non-toxic and harmless solution.	Environmental protection and epidemic prevention: lures and kills mosquitoes using a natural formula that does not harm the human body and surrounding environment.
Human factor design	Integration of greening and mosquito-trapping functions and with a replaceable/addable and removable design.	Establishes epidemic prevention, beautification, and environmental protection for homes and public places in response to diverse needs and enhances urban citizens’ physical and mental balance.

**Table 2:** Proportions of ingredients in mosquito-trapping formula solutions.

	Proportion	High-Proportion Solution	Medium-Proportion Solution	Low-Proportion Solution
Category				
Fermented sugar water Solution		Saturated fermented sugar water solution: 100 g water + 200 g granulated sugar + 2 g yeast powder	Semi-saturated fermented sugar water solution: 100 g water + 100 g granulated sugar + 2 g yeast powder	1/4 saturated solution: 100 g water + 50 g granulated sugar + 2 g yeast powder
Sweet and sour solution		Saturated sweet and sour solution: 100 g water + 200 g granulated sugar + 100 g white vinegar + 2 g yeast powder	Semi-saturated sweet and sour solution: 100 g water + 100 g granulated sugar + 100 g white vinegar + 2 g yeast powder	1/4 saturated sweet and sour liquid: 100 g water + 50 g granulated sugar + 100 g white vinegar + 2 g yeast powder

## Data Availability

All data relevant to this work are included in the article.

## Abbreviation

LLINs, long-lasting insecticidal nets; IRS, indoor residual spraying; LED, light-emitting diode; PET, polyethylene terephthalate; Epoxy Resin AB Glue, epoxy AB; three Dimensions, 3D; acrylonitrile butadiene tyrene, ABS; computerized Numerical Control, CNC; Central Processing Unit, CPU; PCB, printed circuit board.
